# Time-Shift Multi-scale Weighted Permutation Entropy and GWO-SVM Based Fault Diagnosis Approach for Rolling Bearing

**DOI:** 10.3390/e21060621

**Published:** 2019-06-25

**Authors:** Zhilin Dong, Jinde Zheng, Siqi Huang, Haiyang Pan, Qingyun Liu

**Affiliations:** 1 School of Mechanical Engineering, Anhui University of Technology, Ma’anshan 243032, China; 2 Anhui Key Laboratory of Mine Intelligent Equipment and Technology, Anhui University of Science & Technology, Huainan 232001, China; 3 School of Mechanical and Manufacturing Engineering, University of New South Wales, Sydney 2052, Australia

**Keywords:** multi-scale permutation entropy, time-shift multi-scale weighted permutation entropy, rolling bearing, fault diagnosis, gray wolf optimization support vector machine

## Abstract

Multi-scale permutation entropy (MPE) is an effective nonlinear dynamic approach for complexity measurement of time series and it has been widely applied to fault feature representation of rolling bearing. However, the coarse-grained time series in MPE becomes shorter and shorter with the increase of the scale factor, which causes an imprecise estimation of permutation entropy. In addition, the different amplitudes of the same patterns are not considered by the permutation entropy used in MPE. To solve these issues, the time-shift multi-scale weighted permutation entropy (TSMWPE) approach is proposed in this paper. The inadequate process of coarse-grained time series in MPE was optimized by using a time shift time series and the process of probability calculation that cannot fully consider the symbol mode is solved by introducing a weighting operation. The parameter selections of TSMWPE were studied by analyzing two different noise signals. The stability and robustness were also studied by comparing TSMWPE with TSMPE and MPE. Based on the advantages of TSMWPE, an intelligent fault diagnosis method for rolling bearing is proposed by combining it with gray wolf optimized support vector machine for fault classification. The proposed fault diagnostic method was applied to two cases of experimental data analysis of rolling bearing and the results show that it can diagnose the fault category and severity of rolling bearing accurately and the corresponding recognition rate is higher than the rate provided by the existing comparison methods.

## 1. Introduction

Rolling bearing is one of the most important parts of rotating machinery and the one most prone to failure [1]. Due to the complexity of mechanical conditions, once the rolling bearing beings working with failures, it is very likely to cause unpredictable security accidents and economic losses. Thus, it is particularly important to implement condition monitoring and fault diagnosis for rolling bearing [2,3]. Generally, the vibration signals of rolling bearing with failures represent non-linear and non-stationary characteristics and traditional linear or stationary time domain analysis methods have certain limitations when dealing with these types of vibration signals [4,5,6,7]. As a non-linear dynamic analysis tool, entropy plays an important role in measuring complexity and randomness of time series stemming from a nonlinear dynamical system. 

In recent years, lots of nonlinear dynamic methods have been proposed, including approximate entropy (APE) [8], sample entropy (SE) [9], permutation entropy (PE) [10], fuzzy entropy (FE) [11]. The literature [12] indicates that PE at times differed the similar failure modes of complex gearbox vibration signals when it was applied to wind turbine gearbox fault diagnosis. In literature [13], PE was applied to experiment data analysis of rolling bearings and the result concluded that PE is a randomness and dynamic behavior detection method of vibration signals and that it can effectively identify the bearing fault types and degrees. However, the entropy-based indicators mentioned above are generally limited to single-scale analysis of time series and the information of time series on other scales is ignored, which results in a serious loss of information. For this reason, multi-scale sample entropy (MSE) [14], multi-scale fuzzy entropy (MFE) [15] and multi-scale permutation (MPE) were developed by the scholars to measure the complexity of time series in different scales. In literature [16], MSE was applied to rotor fault diagnosis and the experiment results show that MSE contains more information than single-scale sample entropy. In literature [17], MFE was studied and then used to extract the fault features of rolling bearing. However, MSE and MFE also have some intrinsic weaknesses. MPE is an effective method for evaluating the random mutation behavior of time series [18], whose strong anti-noise ability and low computational cost make it stand out. However, due to the limitations of coarse-grained time series, which become shorter and shorter when the scale factor increases, much information of time series is lost. Based on the idea of time-shift coarse-grained time series [19], in this paper, time-shift multi-scale permutation entropy (TSMPE) is developed to enhance the robust performance of MPE, together with the time-shift multi-scale weighted permutation entropy (TSMWPE) based on weighted permutation entropy [20,21]. TSMWPE fully considers the probability calculation of the same modes which have different amplitudes of the state vector in symbol sequence after a reconstruction matrix of coarse-grained time series. TSMWPE optimizes the inadequate coarse-grained time series used in MPE and the process of probability calculation that cannot fully consider the symbol modes. The parameters of TSMWPE are determined by analyzing Gaussian white noise (WGN) and pink noise (1/*f* Noise) data [22] and stability analysis is performed by comparing TSMWPE with TSMPE and MPE. Finally, an intelligent fault diagnosis approach for rolling bearings is proposed based on TSMWPE and gray wolf optimized support vector machine (GWO-SVM) [23] and then applied to two kinds of experimental data analysis of rolling bearing. 

The rest of this paper is structured as follows. In Section 2, the algorithms of MPE and WPE are reviewed and then TSMWPE is developed. The selection of parameters in TSMWPE is studied and the stability analysis among TSMWPE, TSMPE and MPE are made in Section 3. In Section 4, the TSMWPE and GWO-SVM based fault diagnosis approach for rolling bearing is proposed and applied to two sets of experiment data analysis to verify its effectiveness by comparing it with the existing other methods. Finally, Section 5 concludes this paper.

## 2. Algorithm of TSMWPE

### 2.1. MPE method

MPE is proposed to measure the complexity and randomness of time series in multiple scales and its steps can be described as follows.

(1) For a given maximum scale factor $\tau_{\max}$, the coarse-grained time series can be constructed from the original time series {x(i),i=1,2,…,N} by using formula (1)
  (1) $$y_{j}^{\left(\tau \right)}=\frac{1}{\tau }\sum_{i=(j-1)\tau +1}^{j\tau }x_{i},\;\;1\leq j\leq N/\tau$$
  where *j* represents the length of coarse-grained time series.
(2) For the scale factor τ≥2, permutation entropy of each coarse-grained time series is calculated. Finally, the entropy values of all scales are obtained and seen as a function of the scale factor.

The above definition of “coarse-grained” indicates that the length of coarse-grained time series will become short when the scale factor increases, and thus the coarse-grained time series will inevitably cause the important information loss of the original time series.

### 2.2. Algorithm of TSMPE

In this part, TSMPE is firstly developed to solve the problem of MPE mentioned above and its steps can be given as follows.

(1) For a given time series {x(i),i=1,2,⋯,N}, there are
  (2) $$y_{k,\beta }=(x_{k},x_{\beta +k},x_{2\beta +k},\ldots ,x_{\Delta_{\left(\beta ,k\right)}\beta +k})$$
  where the positive integers *k* (1≤k≤τ) and *β* (β=τ), represents the start point and interval of time series. $\Delta_{\left(k,\beta \right)}=\left(N-\beta \right)/k$, indicating the upper boundary number, is a rounded integer.
(2) For scale factor τ≥2, the PEs of each time-shift coarse-grained time series are calculated. The obtained different PEs of each time-shift coarse-grained time series are averaged by
  (3) $$TSMPE(X,\tau ,m,\lambda )=\frac{1}{\tau }\sum_{k=1}^{\tau }\mathrm{TS}PE(y_{k,\beta }^{\left(\tau \right)},m,\lambda )$$
  where *m* is the embedding dimension and λ is delay time.

TSMPE optimizes the insufficient time series coarse granulation process of the MPE algorithm, which makes the time-shift coarse-grained time series have little dependence on the length of the original time series. However, TSMPE does not optimize the process of phase space reconstruction, which undoubtedly makes TSMPE not fully consider the influences of different amplitudes of the same pattern.

### 2.3. Algorithm of TSMWPE

In this subsection, TSMWPE algorithm is proposed to enhance the performance of MPE and TSMPE and its steps can be briefly described as follows.

1. For the original time series {x(i),i=1,2,⋯,N}, the process of time-shift coarse-grained time series $y_{k,\beta }$ can be obtained by Equation (2).
2. For each time-shift coarse-grained time series $y_{k,\beta }$, phase space reconstruction [24,25] is carried out and a matrix (with dimension (n−(m−1)λ)×m) will be constructed.
3. Each row in this matrix is regarded as a state vector and each state vector is mapped into m! possible sorting mode $\pi_{r}$, $f_{\omega }(\pi_{r})$ represents the frequency of the *r*-th permutation in the time series.
  (4) $$f_{\omega }\pi_{r}=\sum_{s=1}^{S}f(\pi_{r}(s))\cdot \omega_{r}(s),s=1,2,\cdots ,S$$
  where *S* is the number of possible patterns in the same motif. If the state vector can be mapped into the sort mode $\pi_{r}$, $f(\pi_{r}(s))=1$ will be obtained, otherwise $f(\pi_{r}(s))=0$. $\omega_{r}$ is denoted as variance of each vector. It represents the weight value for each same pattern, which has different amplitudes.
4. The weighted relative probability of each state vector $p_{\omega }(\pi_{r})$ can be concluded by
  (5) $$p_{w}(\pi_{r})=\frac{f_{\omega }(\pi_{r})}{\sum_{i=1}^{m!}f_{\omega }(\pi_{r})}$$
5. For τ time-shift coarse-grained time series, the weighted permutation entropy of each time-shift coarse-grained time series (TSMPE) can be defined as $H_{w}^{k}$ according to Shannon entropy as
  (6) $$H_{\omega }^{k}=-\sum_{\Pi }^{}p_{\omega }(\pi_{r})\ln p_{\omega }(\pi_{r})\;\;1\leq k\leq \tau$$
6. Finally, τ $H_{w}^{k}$ are obtained and final TSMWPE of original time series is described as
  (7) $$TSMWPE\;(x,\tau ,m,\lambda )=\frac{1}{\tau }\sum_{k=1}^{\tau }H_{w}^{k}(y_{k,\beta },m,\lambda )$$

**Figure 1:**
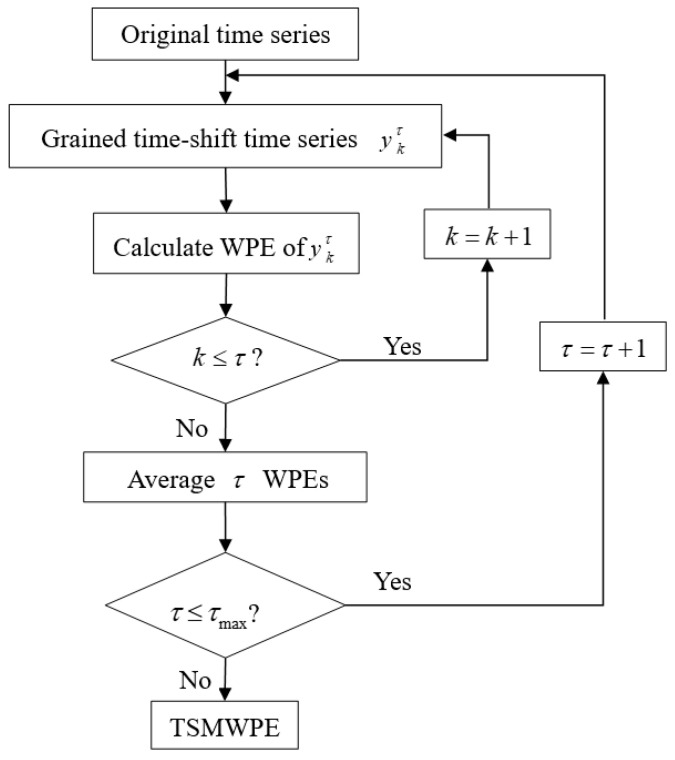
Flowcharts of the TSMWPE method.

Theoretically, TSMWPE relies little on the length of raw signal by applying the idea of time-shift coarse-grained time series. Meanwhile, after the phase space is reconstructed, the probability of symbol sequence using the same pattern but different amplitudes is fully considered through the idea of weighting. The flowchart of the proposed method can be simply described as Figure 1. 

## 3. Analysis of Parameter Selection

### 3.1. Selection of Parameter m

**Figure 2:**
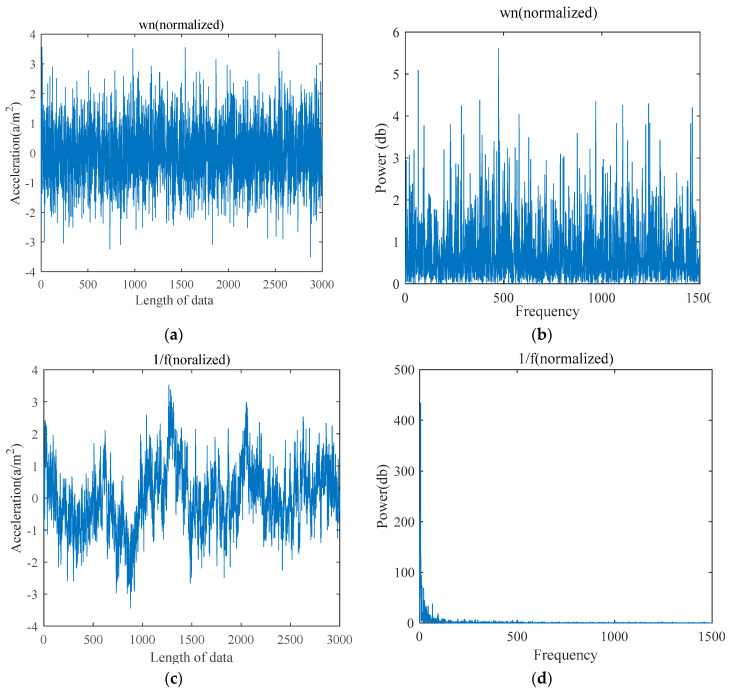
Time domain waveform and power spectrum of WGN and 1/*f* noise. (**a**) Time domain waveform of WGN, (**b**) Power spectrum of WGN, (**c**) Time domain waveform of 1/*f* noise and (**d**) Power spectrum of 1/*f* noise.

The computation of TSMWPE algorithm is affected by the parameters *m*, λ and *N*. If *m* is too small, fewer patterns can be generated by elements contained in the state vector, which will have less significance of the reconstruction matrix. Next, *m* = [4,5,6,7], λ = 1, and *N* = 3000 are selected to study the influence of *m* on TSMWPE. Without the loss of generality, the white Gaussian noise (WGN) and 1/*f* noise are chosen as two examples for experiments and their waveforms in time domain and their power spectrum are shown in Figure 2a–d. 

**Figure 3:**
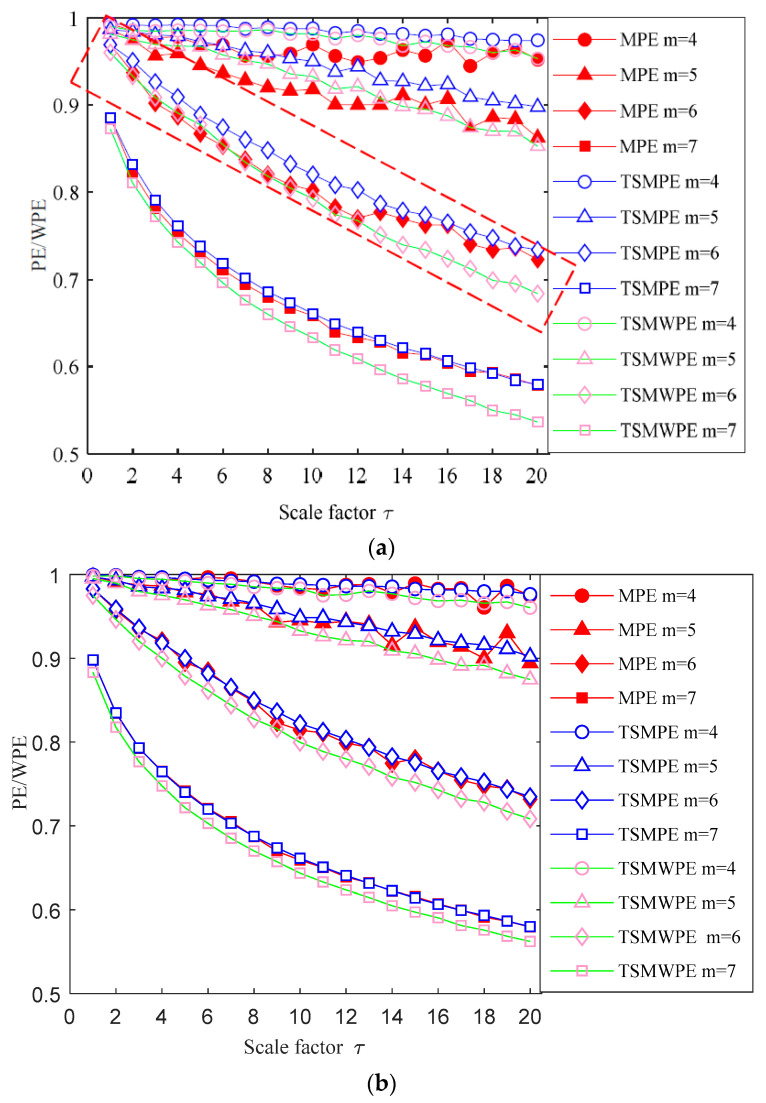
Comparisons of TSMWPE, TSMPE and MPE under different embedding dimensions (**a**) 1/*f* noise and (**b**) WGN.

The comparisons of TSMWPE with TSMPE and MPE are made under different embedding dimensions and the results are shown in Figure 3. From the Figure 3, it can be found that the TSMWPE, TSMPE and MPE values of WGN and 1*/f* noises become smaller and smaller with the increase of *m*. The TSMWPE, TSMPE and MPE curves for different embedding dimensions are compared in Figure 3a. It can be found that when *m* = 4, TSMWPE, TSMPE and MPE have larger entropy values from a scale of 1 to 20. It has a small range of variations from 0.95 to 1. The result indicates the smaller the value of parameter *m*, the fewer types of patterns are produced by each state vector. Even though the occurrence number of state vectors with the same pattern but different amplitudes is relatively small, the occurrence frequency is relatively larger, which causes an increase in the weighted relative probability of each pattern. Eventually the calculated entropy value will inevitably become larger. When *m* = 6 or 7, there is a large ranges of amplitude changes, which indicates that it can more fully reflect the change process of entropy value on different scale factors relatively to when *m* = 4 or 5. However, when *m* = 7, it can be found that there is a great consistency between TSMPE and MPE, and it is difficult to distinguish between MPE and TSMPE. Based on the analysis above, the parameter *m* = 6 is chosen in TSMWPE, TSMPE and MPE. In addition, it is worth noting that the curve of TSMWPE is significantly more stable than that of MPE, which indicates that TSMWPE exhibits good stability in feature extraction. 

### 3.2. Selection of Parameter λ

**Figure 4:**
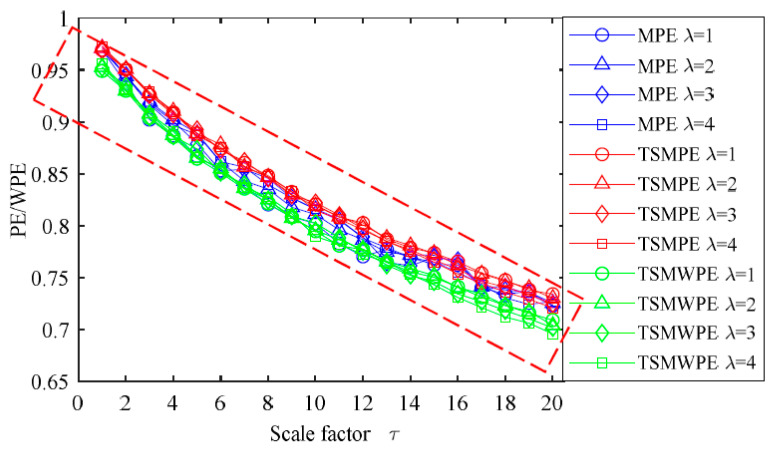
Comparisons of TSMWPE, TSMPE and MPE under different time delay of 1/*f* noise.

Based on the analysis above, the effect of *m* on TSMWPE, TSMPE and MPE curves is studied and *m* is set as 6 in the following part. The delay time λ = [1,2,3,4] and the 1/*f* noise with a length of 3000 is chosen to analyze the effect of different delay time on TSMWPE. The TSMWPE, TSMPE and MPE of 1/*f* noise signals under different time delays are computed and shown in Figure 4. From Figure 4 the linear trend of TSMWPE is consistent with that of TSMPE and MPE. The entropy values are very close for different time delays. Therefore, the time delay generally has a very slight effect on TSMWPE and thus the delay time λ is set as 1. In addition, it can be found that the TSMWPE and TSMPE curves are smoother than MPE, because the process of time-shift coarse-grained time series saves more useful time information and can effectively improve the process of a single coarse-grained time series.

### 3.3. Selection of Parameter N

**Figure 5:**
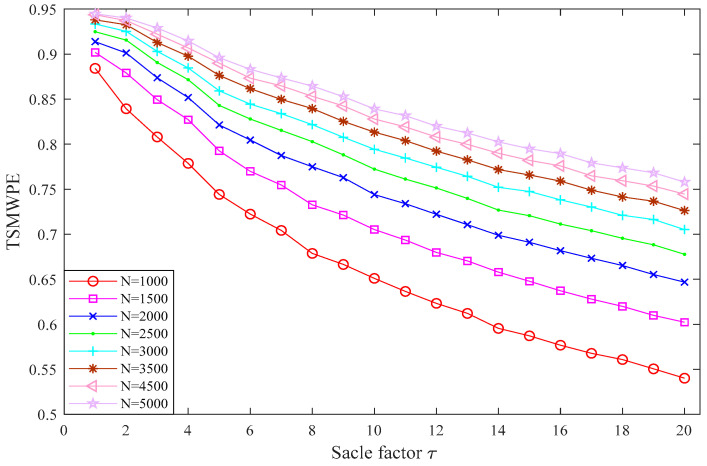
TSMWPE of 1/*f* noise under different length *N*.

In this subsection, the 1/f noise with lengths of *N* = 1000, 1500, 2000, 2500, 3000, 3500, 4000, 4500 are set with *m* = 6 and time delay λ=1 to determine the effect of parameter *N* on TSMWPE. The TSMWPE of 1/*f* noise with different lengths are shown in Figure 5. It can be found from Figure 5 that the TSMWPE curve of 1/*f* noise shows a slight fluctuation when *N* ≤ 2500 and when *N* ≥ 2500, it appears to be quite stable and has a nearly parallel trend. Therefore, *N* ≥ 3000 is generally selected in the subsequent step.

### 3.4. Stability Analysis

**Figure 6:**
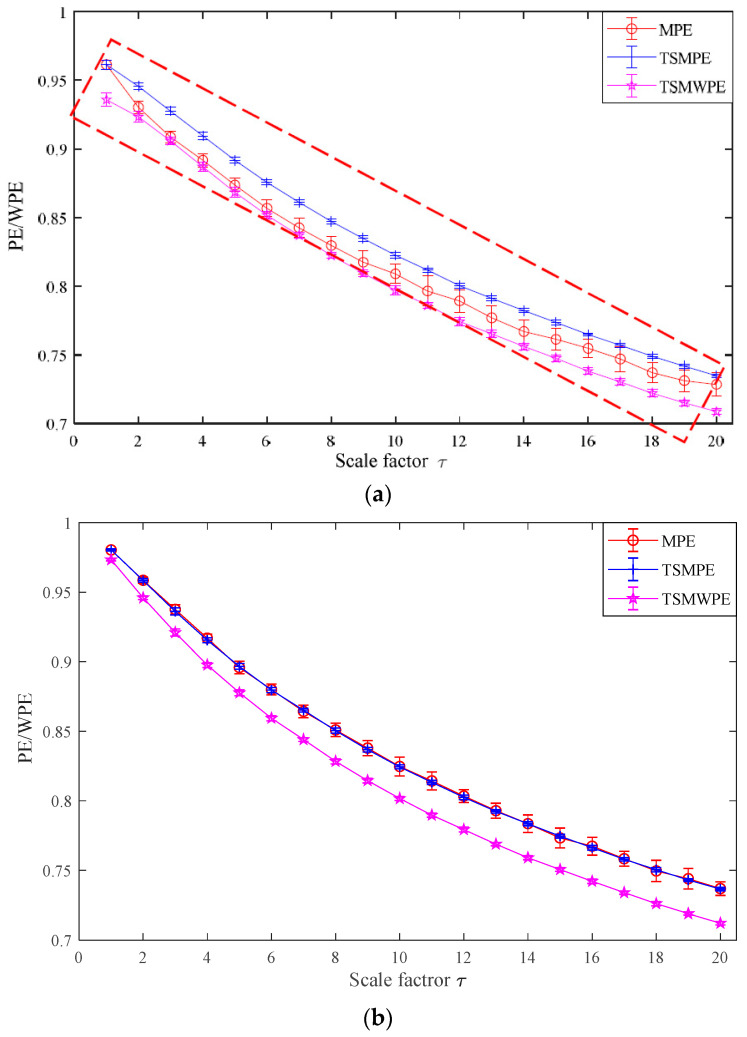
The mean standard deviation of MPE/TSMPE/TSMWPE under the same parameter (**a**) 1/*f* noise and (**b**) WGN.

Based on the analysis above, we set *m* = 6 and λ=1. 20 sets of WGN and 1/f noise with a length of 3000 are selected to verify the superiority of TSMWPE in feature extraction. The mean standard deviations of TSMWPE, TSMPE and MPE under the same parameter are shown in Figure 6. It can be seen from the Figure 6 that for WGN and 1/*f* noise, the standard deviations of TSMWPE and TSMPE are much smaller than that of MPE, which indicates that TSMPE and TSMWPE are more stable than MPE in feature extraction. The standard deviations of TSMWPE are not much different from that of TSMPE, and the curve of TSMWPE is relatively smooth and stable. 

## 4. TSMWPE and GWO-SVM Based Fault Diagnosis Method for Rolling Bearing

### 4.1. GWO-SVM

Generally, the parameters in original SVM were set by the users’ experience. Once the kernel function has been selected, it will not be changed, This inevitably makes the classification effect of SVM be limited. Therefore, it is necessary to study the selection of kernel functions and setting of parameters [26]. In this paper, the penalty factor c and the parameter g of radial basis kernel function of SVM are optimized by the gray wolf optimization algorithm.

The GWO algorithm was proposed by Seyedai et al [27] in 2014 inspired by the division of labor between wolves and collaborative hunting of food. It is a new swarm intelligence algorithm that simulates the hierarchy in wolves and the hunting behavior of wolves. Followed by wolf species B, wolf species C and wolf species E, the highest ranking wolf is the wolf species A, which is located at the top of the food chain and is responsible for leadership, decision making and other behaviors. Although wolf species B and wolf species C are not the highest-ranking wolf species, they can succeed wolf species A and become a new leader when the wolf species A loses its leadership. Wolf species E, the lowest level of wolf, is responsible for balancing the relationship between the inside of the population.

The GWO algorithm treats each wolf as a potential solution, where wolf species A is the first optimal solution, while wolf species B and C are respectively the second optimal solution and the sub-optimal solution. The GWO algorithm is an iterative optimization process in which the positions of wolves A, B and C are constantly updated. The wolves update the distance and position through the formulas (8) and (9) to complete the search for the prey.

(8) $$D=|C\times X_{p}(t)-X(t)|$$

(9) $$X(t+1)=X_{p}(t)+D$$

where *D* is the distance between gray wolf and the prey, *t* is the number of iterations; $X_{p}$ indicates the position of the prey, X indicates the position of the gray wolf and its initial position coordinates are defined as (*c*, *g*). *A* and *C* represent the coefficients where $A=2a\times r_{2}-a$, $C=2r_{1}$. When |A|>1 it represents a global search, that is, the gray wolf group expands the search range to find a better prey. In contrast, while |A|≤1, it represents a local search, and the gray wolf group will narrow the encirclement and search for the prey nearby. $a=2-\frac{2t}{t\max}$ and the convergence factor *a* linearly decreases from 2 to 0 as the number of iterations increases, and $t_{\max}$ is the maximum number of iterations. $r_{1}$ and $r_{2}$ are respectively a random value of [0,1].

When the gray wolf judges the position of the prey, the head wolves A lead the wolves B and wolves C to surround the prey, because wolves A, B, and C are the closest to the prey, so the position of the three wolves gradually approaches the prey, they are described as follows.

(10) $$D_{a}=|C_{1}\times X_{a}(t)-X(t)|$$

(11) $$D_{b}=|C_{2}\times X_{b}(t)-X(t)|$$

(12) $$D_{c}=|C_{3}\times X_{c}(t)-X(t)|$$

where X*_a_* represents current location of wolves A, X*_b_* represents current location of wolves B, $X_{c}$ represents current location of wolves C. $C_{1}$, $C_{2}$ and $C_{3}$ are random variables. X(t) is the current location of the wolf species. The step lengths and directions of wolves E to wolves A, B and C are defined by formulas (13)–(15) and the final position of wolves E are defined by formulas (16).

(13) $$X_{1}=|C_{3}\times X_{a}-A_{1}\times D_{a}|$$

(14) $$X_{2}=|C_{2}\times X_{b}-A_{2}\times D_{b}|$$

(15) $$X_{3}=|C_{3}\times X_{c}-A_{2}\times D_{c}|$$

(16) $$X_{\left(t+1\right)}=\left|\frac{\left(X_{1}+X_{2}+X_{3}\right)}{3}\right|$$

**Figure 7:**
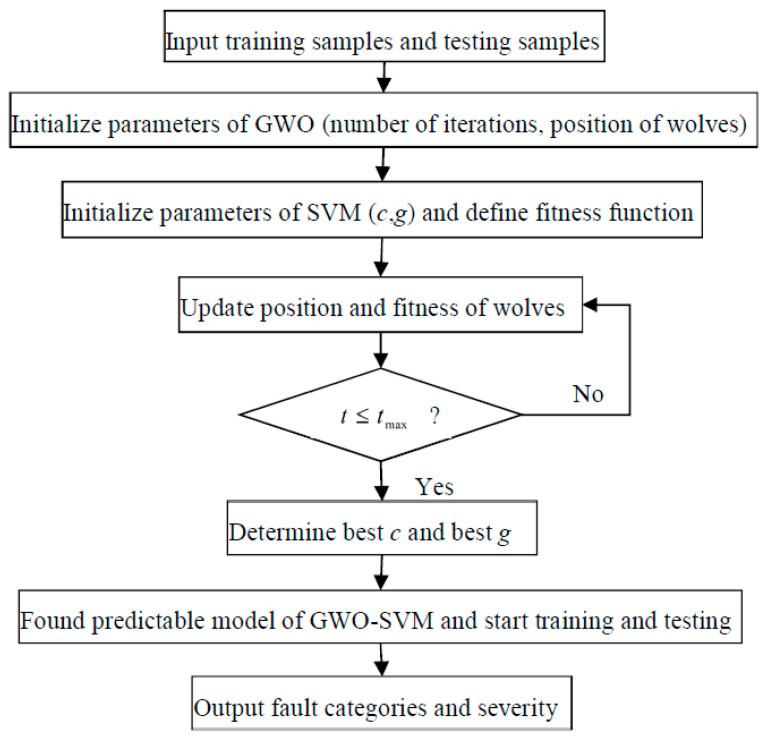
The flowchart of GWO-SVM.

When the wolves are hunting, wolves A, wolves B and wolves C have different fitness values for the prey. By calculating different fitness values, the first optimal solution, the optimal solution and the sub-optimal solution are obtained, and the current position information is saved. Meanwhile, the wolves judge the moving direction of the prey and approach the prey to complete the hunting based on the three sets of positional information. After that, the positions of the gray wolves are updated again until the first optimal solution is provided. The position coordinate value corresponding to the first optimal solution is defined as (best *c*, best *g*). The flowchart of GWO-SVM is shown in Figure 7. The GWO-SVM can optimize the penalty factor *c* and the parameter *g* in kernel function of original SVM and ensure that the best *c* and the best *g* can be found, which is more superior to SVM in theory.

The GWO-SVM can optimize the penalty factor *c* and the parameter *g* in the kernel function of the original SVM and ensure that the best *c* and the best *g* can be found, which is superior to SVM in theory. The best *c* and the best *g* change as different models change.

### 4.2. The Proposed Fault Diagnosis Approach

Due to the advantages of TSMWPE and GWO-SVM, the GWO-SVM based multi-class classifier is constructed to achieve an intelligent fault diagnosis of rolling bearing. The steps of the proposed methods for rolling bearing can be described as follows.

**Figure 8:**
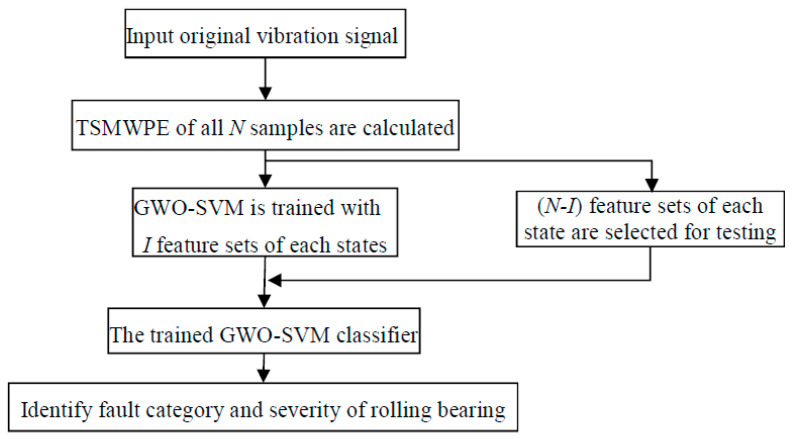
Flowchart of the proposed fault diagnosis approach.

(1) Let the rolling bearing contains *K* class work conditions, *N* sets of samples are collected for each state. TSMWPE is computed for all samples of each state of rolling bearing in *M* scales. The TSMWPE values obtained are used as the sample feature information to form the original feature vector matrix $R^{K\times N\times M}$.
(2) For each state of rolling bearing, *N* samples are collected and *I* samples are selected from the N ones as training samples to form a feature training set ($R^{K\times I\times M}$) and the rest (*N*−*I*) ones are seen as testing samples to form the testing feature set ($R^{K\times (N-I)\times M}$).
(3) The training model feature set is employed to train the GWO-SVM based multi-classifier.
(4) The testing sample feature set is inputting to the trained multi-classifier for prediction. The fault categories and severity of rolling bearing are judged according to the output of GWO-SVM multi-fault classifier. The flowchart of proposed method of fault diagnosis is shown in Figure 8.

### 4.3. Experimental Analysis of Rolling Bearing

#### 4.3.1. Case 1

**Figure 9:**
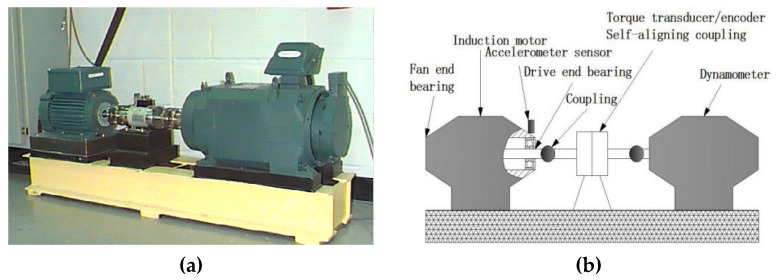
The test rig of CWRU (**a**) The test rig of CWRU and (**b**) its sketch.

**Figure 10:**
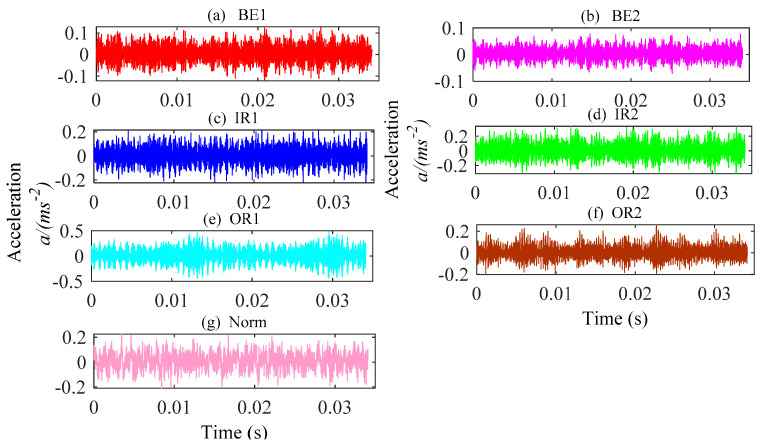
The waveforms of vibration signals of rolling bearings for case 1. (**a**) BE1; (**b**) BE2; (**c**) IR1; (**d**) IR2; (**e**) OR1; (**f**) OR2; (**g**) Norm.

In this subsection, the experimental data of Case Western Reserve University [28] are used to verify the proposed method in fault diagnosis of rolling bearing. As shown in Figure 9, the test rig consists of a fan end bearing, a drive end bearing and a torque transducer. The type of tested rolling bearing is 6205-2RS JEM SKF deep groove ball bearing; here single point faults are seeded to the rolling bearings through electric discharge machining technology. In the test, the rotary speed is 1, 730 r/min, load of rolling bearing is 2205 W and sampling frequency is 12 kHz. The data with fault diameters 0.5334 mm and 0.1778 mm are applied in the following part. The vibration signals are collected from normal (Norm) and inner race (IR) Ball Element (BE) and Outer Race (OR) with local single point pitting and they are successively denoted in Table 1. Experimental data is collected by the acceleration sensor in which the binary counting method is adopted. Each class has 20 samples with the length of 4096 points and the waveforms of vibration signal of rolling bearings are shown in Figure 10.

**Figure 11:**
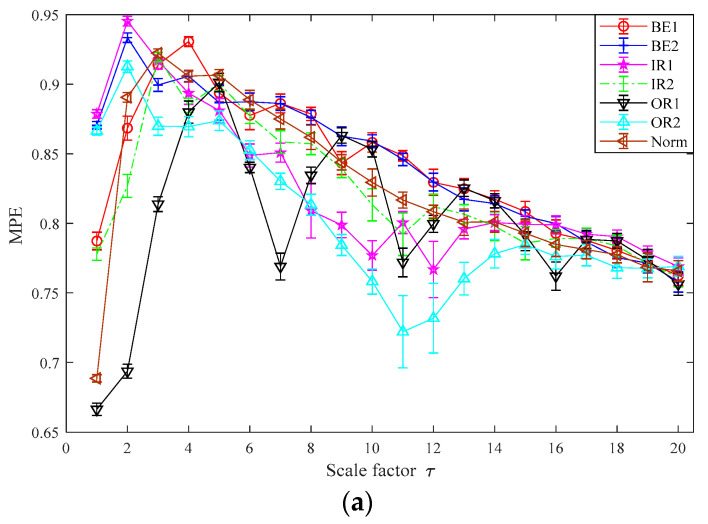

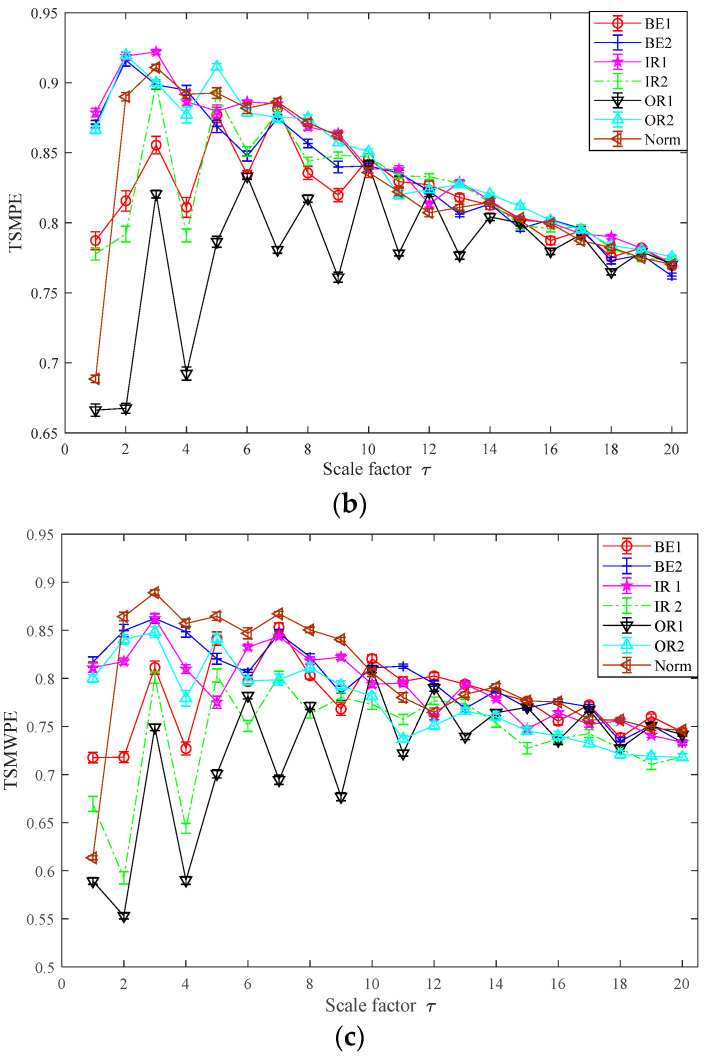
MPE, TSMPE and TSMWPE of different states of rolling bearings for case 1 (**a**) MPE (**b**) TSMPE and (**c**) TSMWPE.

In this part, the TSMWPE, TSMPE and MPE values of 140 samples of rolling bearing with seven fault categories and degrees are calculated with the parameter *m* = 6 and λ=1. The mean values and standard deviations of TSMWPE, TSMPE and MPE for all samples are depicted in Figure 11a–c. First, it can be concluded from Figure 11 that the TSMWPE, TSMPE and MPE trends of fault vibration signals gradually decrease with the increase of the scale factor. Second, the trends of TSMPE and TSMWPE are very similar, but they are different from that of MPE, especially for BE1 and OR1. Third, TSMWPE is more stable than MPE under all states of rolling bearings especially for OR2. It is very difficult to judge the superiority of TSMWPE by observing the curve of TSMWPE, TSMPE and MPE. The fault identification accuracy will be compared by combining TSMWPE, TSMPE, MPE with GWO-SVM for fault feature extraction and classification. The seven states of rolling bearing are marked as 1 to 7. Among the 20 samples of each class, 10 samples are randomly selected from all ones and seen as training data, while the remaining 10 are used for testing. 

**Figure 12:**
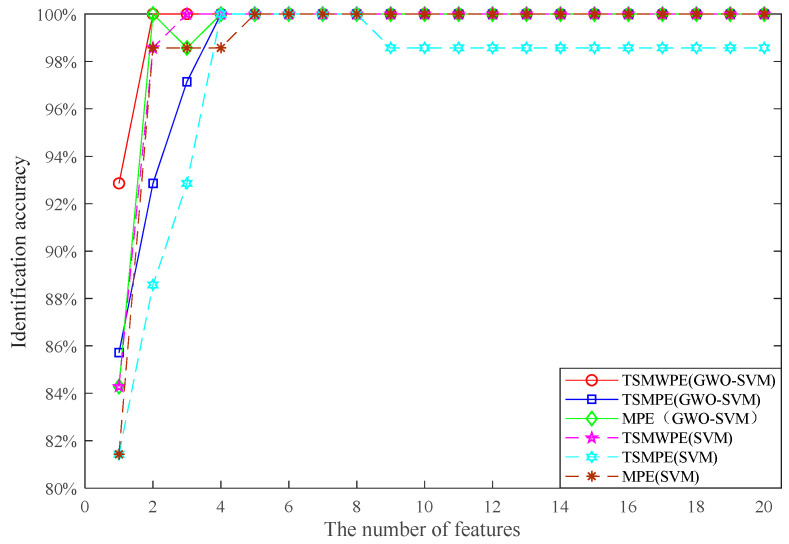
Comparison of identification accuracy for different number of features.

In order to explore the superiority of TSMWPE to TSMPE and MPE, the TSMWPE, TSMPE and MPE of all samples are computed to make a comparison analysis. Then the first *d* (*d* = 1, 2, …, 20) TSMWPE to TSMPE and MPE values are taken as fault features and used to train the GWO-SVM classifier, where the optimized parameters are shown in Table 2, Table 3 and Table 4. It can be seen from the Table 2, Table 3 and Table 4 that when the number of used features is different, the corresponding best *c* and best *g* are different. That indicates that according to the status of feature input, the best *c* and the best *g* will make corresponding changes to optimizing the Performance of SVM. The corresponding identification accuracy for different number of features is shown in Figure 12. It can be seen from the Figure 12 that when the single feature, i.e., only WPE is used, the recognition accuracy of TSMWPE and GWO-SVM based fault diagnosis method of rolling bearing is 92.8%. The recognition accuracy of proposed method will maintain at 100% when the number of inputting features is larger than one. The recognition accuracy of MPE and GWO-SVM based fault diagnosis method are correspondingly 84.3% and 98.6% when the single feature and the first three features are used. When the first, the first two and the first three TSMPE features are input into the trained GWO-SVM classifier, the identification accuracy of GWO-SVM classifier are 85.7%, 92.9% and 97.1%. Also, the original un-optimized SVM is used for comparison to verify the necessity and superiority of GWO-SVM, where the kernel function used in SVM is polynomial function. It can be seen from Figure 12 that the identification accuracy of TSMWPE and SVM based fault diagnosis method is 85.7% and 98.6% when the single feature and the first two features are used and always remains at 100% after there are more than three inputting features. However, by observing Figure 12, it can be found that for the equal number of inputting features (less than five), the highest fault identifying accuracy are generally obtained by the proposed method rather than other methods. Therefore, we set the number of inputting number ranging from 5 to 10 for a high and fast diagnosis. And the above analysis also indicates that TSMWPE is an effective method for distinguishing the fault categories and degrees of rolling bearings. 

#### 4.3.2. Case 2

**Figure 13:**
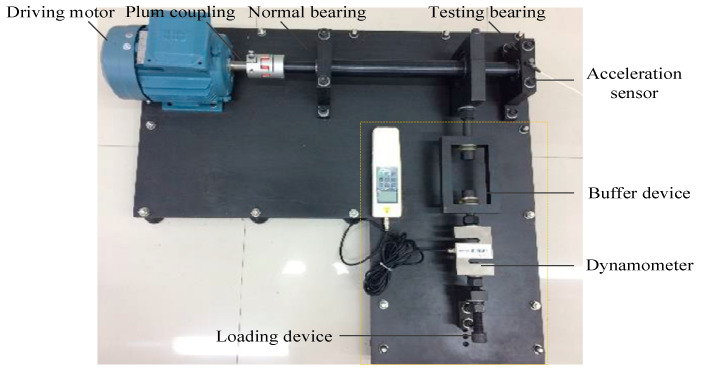
The rolling bearing test rig of Soochow university for case 2.

**Figure 14:**
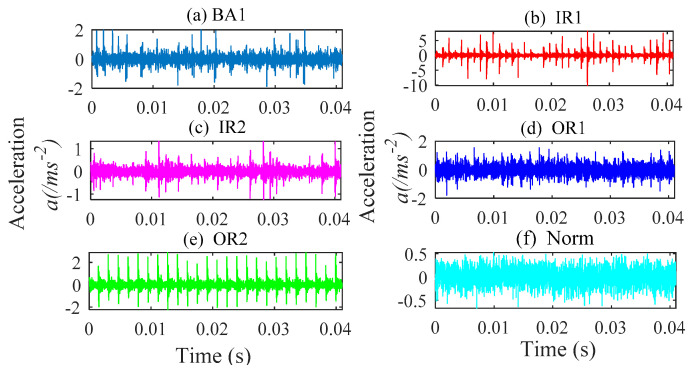
Waveforms of vibration signal of rolling bearing from case 2. (**a**) BA1; (**b**) IR1; (**c**) IR2; (**d**) OR1; (**e**) OR2; (**f**) Norm.

The experimental data of rolling bearing used in this subsection were provided by Soochow University [29,30] to further verify the effectiveness of the proposed method. The test bearing is 6205-2RS deep groove ball bearing and the faulty rolling bearings are machined by a metal electric engraving machine to set a local fault. The spindle speed is 900 r/min and sampling frequency is 10 kHz. The experiment has six different fault classes and locations of rolling bearings, which are listed in Table 5. The test rig of rolling bearing is shown in Figure 13. The operating system includes plum coupling, driving motor, testing bearing, normal bearing, acceleration sensor, buffer device, dynamometer and loading device. Each class of rolling bearing has 28 samples with a length of 4096 points and the vibration signal waveforms of rolling bearing are depicted in Figure 14. 

**Figure 15:**
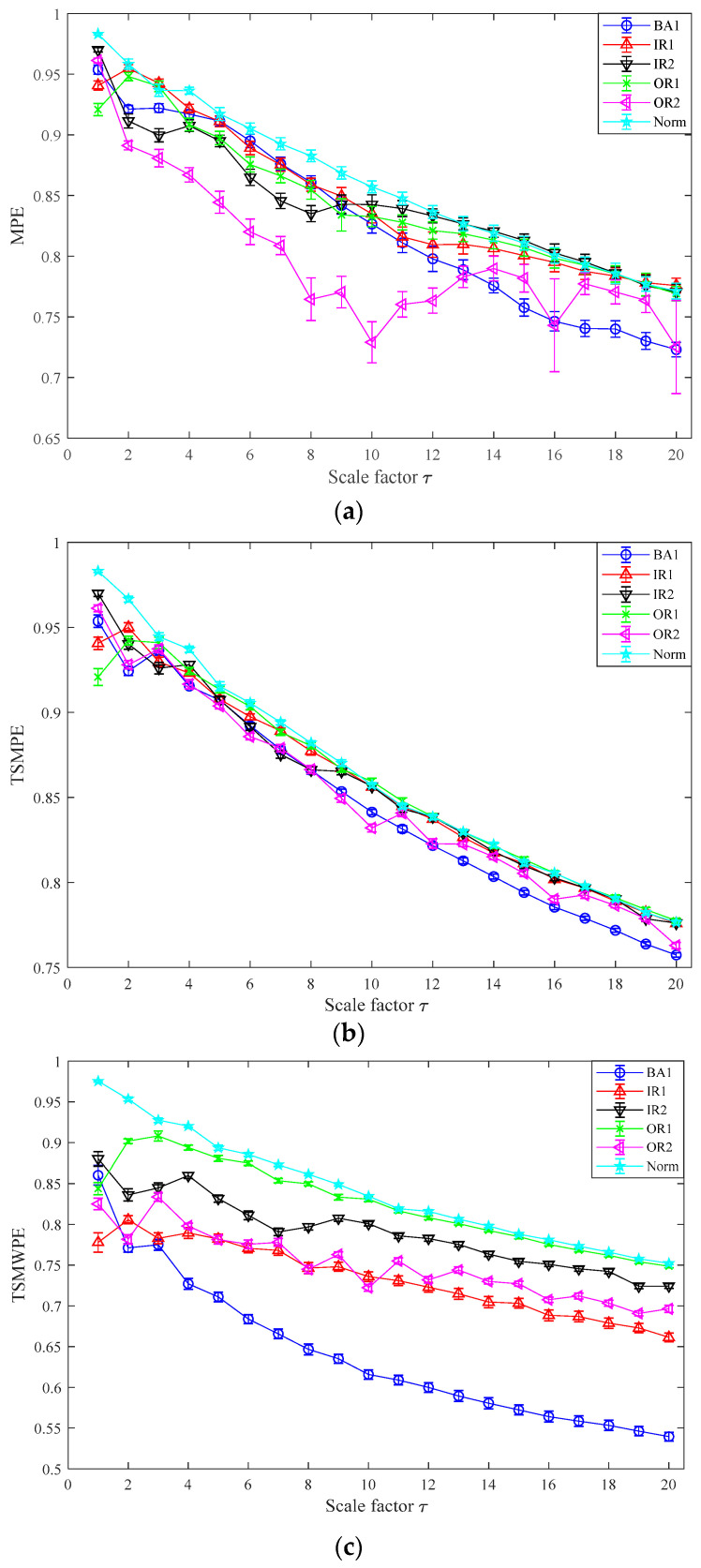
MPE, TSMPE and TSMWPE of different states for rolling bearings for case 2. (**a**) MPE; (**b**) TSMPE; and (**c**) TSMWPE.

The TSMWPE, TSMPE and MPE of all 28 samples of each class are calculated with the parameter *m* = 6, λ=1, and the mean values and standard deviations are shown in Figure 15a–c. First, it can be obviously obtained from Figure 15 that the standard deviation of MPE is larger than that of TSMPE and TSMWPE, especially for OR2. Second, the TSMPEs are much denser than TSMWPEs and MPEs, while TSMWPEs are more scattered than TSMPEs and MPEs. The above analysis indicates that the TSMWPE based feature extraction method has irreplaceable superiority to MPE and TSMPE and is more stable and robust than TSMPE and MPE.

**Figure 16:**
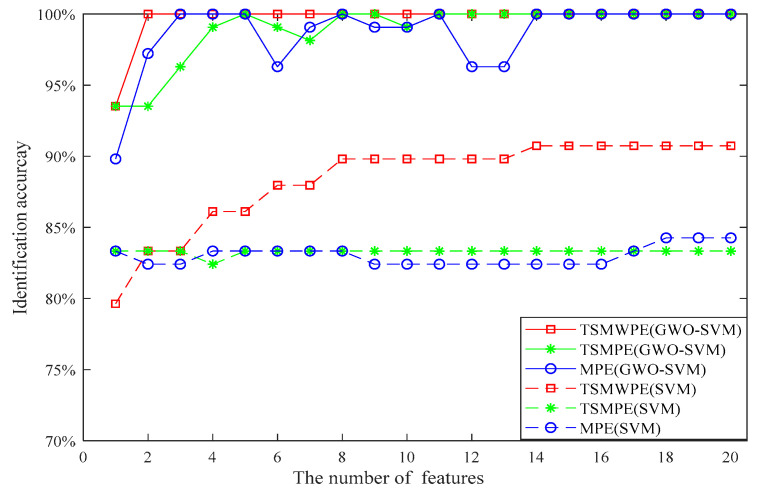
Comparison of identification accuracy for different number of features. (GWO-SVM and SVM).

Next, 10 samples of each class rolling bearing are randomly chosen from 28 samples for training and the left 18 are used for testing. Therefore, the fault features (with dimensions 60 × 20) can be obtained and employed to train the GWO-SVM based multi-classifier and the left fault feature sets (with dimensions 108 × 20) are inputting to the trained multi-classifier for testing. The identification accuracy of the proposed method with different numbers of inputting features used are given in Figure 16, together with that of the TSMPE and MPE based methods, where the optimized parameters in GWO-SVM for the three methods are shown in Table 6, Table 7 and Table 8. It can be seen from Figure 16 that for the TSMWPE and GWO-SVM based fault diagnosis method, the recognition accuracy when the single feature is considered is 93.5% and it remains at 100% when the number of features is larger than 2. In fact, the identification accuracy of the proposed method is higher than that of MPE and TSMPE based methods for different numbers of features. 

The GWO-SVM in the proposed method was also replaced by original SVM for comparison. Like the above process, the identification accuracy of the TSMWPE, TSMPE and MPE based fault extraction method by combing SVM for classification is given in Figure 16. It can be observed from Figure 16 that for the first eight features, the identification accuracy of the TSMWPE and SVM based method gradually increases from 79.6% to 89.8% then remains at 89.8% from the eighth features to the fourteenth features and at 90.7% from the fourteenth features to the twentieth feature. The identification accuracies of the fault diagnosis method based on TSMPE and SVM are stable at 83.3% when the number of features is larger than 4. The identification accuracy of the MPE and SVM based fault diagnosis method varies from 82.4% to 84.3%. The identification accuracy of GWO-SVM based fault classification is higher than 90%, while that of SVM based multi-classifier is lower than 90% and this indicates the superiority of GWO-SVM to SVM. By observing Figure 16 carefully, it can be found that the GSOSVM is superior to SVM and the proposed TSMWPE and GWO-SVM based fault diagnosis method has higher fault identifying rates than other comparative methods. Generally, we set the number of input number ranging from 5 to 10 for a high diagnosis effect. Therefore, the results above demonstrate the superiority of TSMWPE to TSMPE and MPE in feature extraction, together with that of GWO-SVM to original SVM.

## 5. Conclusions

In this paper, the TSMWPE algorithm was proposed to measure the complexity and irregularity of time series, which can effectively optimize the traditional coarse-grained time series and fully consider the same symbol modes with different amplitudes, in which the weighted relative probability of each pattern is calculated. Also, the superiority of TSMWPE to MPE and TSMPE was further verified by two simulation analyses. Based on TSMWPE and GWO-SVM, a new fault diagnosis method for rolling bearing was proposed and applied to two experimental data case analyses of experiment data of rolling bearing. The proposed fault feature extraction method of rolling bearing was compared with MPE and TSMPE based fault feature extraction one and the analysis results validated that TSMWPE shows a better performance than MPE and TSMPE, and the TSMWPE and GWO-SVM based fault diagnosis method has a higher recognition accuracy than the TSMPE and GWO-SVM based method, together with the MPE and GWO-SVM based method. Also, the GWO-SVM for fault classification method was compared with the original SVM to verify the effectiveness of the proposed method. Additionally, the number of inputting features were discussed and recommended in the paper. In future work, the TSMWPE algorithm will be further studied and applied to machine condition monitoring. 

## Figures and Tables

**Table 1:** The types and degrees of faulty rolling bearing for case 1.

Abbreviation	Fault Location	Fault Diameter (mm)
BE1	Ball element	0.1778
BE2	Ball element	0.5334
IR1	Inner race	0.1778
IR2	Inner race	0.5334
OR1	Outer race	0.1778
OR2	Outer race	0.5334
Norm	Normal bearing	0

**Table 2:** The best *c* and the best *g* in TSMWPE and GWO-SVM based fault diagnosis method for case 1.

Number of Used Features	1	2	3	4	5	6	7	8	9	10
Best *c*	2.7	19.3	81.1	12.7	4.9	14.5	90.4	34.5	3.6	52.8
Best *g*	67.8	14.1	35.4	26.7	33.7	5.4	46.8	92.7	33.7	16.9
**Number of used features**	11	12	13	14	15	16	17	18	19	20
Best *c*	58.4	48.3	91.5	6.2	83.5	48.2	68.2	91.9	22.1	5.1
Best *g*	1.1	20.0	0.6	8.1	19.9	15.2	17.6	0	24.5	0

**Table 3:** The best *c* and the best *g* in TSMPE and GWO-SVM based fault diagnosis method for case 1.

Number of Used Features	1	2	3	4	5	6	7	8	9	10
Best *c*	72.6	64.1	70.6	1.0	55.3	48.7	7.9	66.6	43.4	49.0
Best *g*	100	15.9	14.0	57.6	46.6	77.0	64.8	10.4	20.5	2.9
**Number of used features**	11	12	13	14	15	16	17	18	19	20
Best *c*	34.1	58.1	63.6	97.2	40.0	52.8	93.7	14.0	79.9	61.5
Best *g*	2.8	36.8	13.5	7.0	10.2	16.9	17.6	5.5	6.9	1.3

**Table 4:** The best *c* and the best *g* in MPE and GWO-SVM based fault diagnosis method for case 1.

Number of Used Features	1	2	3	4	5	6	7	8	9	10
Best *c*	36.4	78.2	29.6	94.4	3.8	34.1	84.5	3.1	62.4	37.9
Best *g*	97.1	38.7	70.0	55.2	14.9	9.1	13.9	8.2	0.0	16.3
**Number of used features**	11	12	13	14	15	16	17	18	19	20
Best *c*	17.9	31.6	72.2	0.0	55.8	89.0	73.4	14.6	92.4	2.3
Best *g*	11.4	8.9	13.5	0.0	0.0	0.0	0.3	0.2	6.7	0.0

**Table 5:** The fault locations and degrees of faulty rolling bearing for case 2.

Abbreviation	Fault Location	Fault Diameter (mm)
BE1	Ball element	0.6
IR1	Inner race	0.2
IR2	Inner race	0.6
OR1	Outer race	0.2
OR2	Outer race	0.6
Norm	Normal bearing	0

**Table 6:** The best *c* and the best *g* in TSMWPE and GWO-SVM based fault diagnosis method for case 2.

Number of Used Features	1	2	3	4	5	6	7	8	9	10
Best *c*	42.9	97.3	19.1	29.8	53.6	86.7	39.3	41.1	67.0	15.8
Best *g*	54.1	96.8	87.3	26.5	62.9	34.9	3.3	60.7	3.1	33.7
**Number of used features**	11	12	13	14	15	16	17	18	19	20
Best *c*	17.3	45.3	24.9	45.2	98.5	58.2	29.7	40.1	33.7	97.8
Best *g*	82.6	51.5	32.5	2.6	59.8	64.4	85.5	38.6	33.6	21.2

**Table 7:** The best *c* and the best *g* in TSMPE and GWO-SVM based fault diagnosis method for case 2.

Number of Used Features	1	2	3	4	5	6	7	8	9	10
Best *c*	78.4	16.8	100	15.3	15	68.6	36.7	96.2	68.1	89.1
Best *g*	31.2	13.7	23.8	29.4	38.4	0.0	30.8	0.9	89.6	88.5
Number of used features	11	12	13	14	15	16	17	18	19	20
Best *c*	69.8	98.4	68.4	31.4	24.6	58.7	91.5	90.9	15.7	37.9
Best *g*	61.6	26.8	83.8	83.0	33.1	42.1	85.5	36.3	75.1	89.3

**Table 8:** The best *c* and the best *g* in MPE and GWO-SVM based fault diagnosis method for case 2.

Number of Used Features	1	22	3	4	5	6	7	8	9	10
Best *c*	29.8	18.3	4.6	63.8	48.9	0.3	0.0	48.6	43.3	57.4
Best *g*	95.0	40.0	3.1	6.1	18.4	2.5	4.8	9.0	1.7	19.5
Number of used features	11	12	13	14	15	16	17	18	19	20
Best *c*	58.4	13.6	100.0	25.7	17.4	70.0	60.0	77.4	79.1	55.1
Best *g*	0.0	4.2	7.7	1.7	0.0	4.7	3.6	9.9	2.1	3.7
